# Pollen Morphology of Convolvulaceae from Southeastern Amazonian Cangas and Its Relevance for Interaction Networks and Paleoenvironmental Studies

**DOI:** 10.3390/plants12122256

**Published:** 2023-06-09

**Authors:** Luiza de Araújo Romeiro, Edilson Freitas da Silva, Liziane Vilela Vasconcelos, Karen da Silva Lopes, Léa Maria Medeiros Carreira, José Tasso Felix Guimarães

**Affiliations:** 1 Instituto Tecnológico Vale, Rua Boaventura da Silva 955, Nazaré, Belém 66055-090, PA, Brazil; luizaromeiro84@gmail.com (L.d.A.R.); freitasdasilva20@yahoo.com.br (E.F.d.S.); karen.lopes@outlook.com (K.d.S.L.); 2 Programa de Pós-Graduação em Ecologia, Universidade Federal do Pará, Rua Augusto Corrêa 01, Guamá, Belém 66075-110, PA, Brazil; lizianevilela@gmail.com; 3 Museu Paraense Emílio Goeldi, Coordenação de Botânica, Avenida Perimetral 1901, Terra Firme, Belém 66077-830, PA, Brazil; lea@museu-goeldi.br

**Keywords:** pollen morphology, Convolvulaceae, Serra dos Carajás, endangered plants, canga vegetation

## Abstract

Serra dos Carajás harbors a unique open plant community in Amazonia, known as *canga* vegetation, with several endemic species coexisting with the potential threat of large-scale iron ore mining. In this sense, Convolvulaceae occur in a wide variety of canga geoenvironments with multiple flower visitors, but the scarcity of data on its pollen morphology prevents the correct association between Convolvulaceae species with floral visitors, as well as the precise identification of their habitats throughout the Quaternary. Therefore, this study aims to contribute to the taxonomic knowledge and refinement of the identification of insect-plant networks of endangered plants, including *Ipomoea cavalcantei*. Pollen grains were examined by light and scanning electron microscopy (LM and SEM, respectively), and the morphological parameters obtained were statistically analyzed using principal component analysis. Therefore, all species were differentiated based on aperture types and exine ornamentation. The set of morphological characters indicated that echinae morphology, easily identified under LM, was effective for the identification of *Ipomoea* species. This work represents the first robust pollen database for a precise identification at the species level of Convolvulaceae from southeastern Amazonian cangas.

## 1. Introduction

Convolvulaceae Juss. comprises approximately 1900 species distributed in 60 genera practically spread all over the world over a broad range of habitats, such as perennial herbs, vines, woody lianas, shrubs, or trees that are endemic to tropical regions [1,2,3,4,5,6]. In Brazil, 24 genera and approximately 420 species are recognized, occurring in various vegetation formations [7,8]. *Daustinia* Buril & A.R.Simões is the only genus endemic to the Flora of Brazil, where [4] transferred a Brazilian species of Jacquemontia Choisy to this new genus [4,9].

Serra dos Carajás, southeastern Amazonia, harbors approximately 30 Convolvulaceae species distributed in nine genera, with 17 of these species occurring exclusively in ironstone outcrops [10], known as canga, that is surrounded by dry and humid evergreen tropical forests [11,12]. Convolvulaceae is highly represented in canga vegetation, predominantly herbaceous and shrubby, associated with outcrops of ferruginous rocks, thus presenting a wide variety of geoenvironments, such as rocky and hydromorphic fields, as well as forest for-mations [12]. The extreme conditions in which the cangas are inserted, such as: acidic soils, poor in nutrients, in addition to high temperatures and strong seasonality, provide an environmental peculiarity, for the occurrence of a large number of endemic and rare species, among them *Ipomoea cavalcantei* D.F. Austin [12,13].

Indeed, even the Convolvulaceae pollen are common on bee honeys [14], lake surface sediments [15,16], and Quaternary lake cores [17,18]. However, these studies unfortunately made a genus-level identification. This makes it difficult to develop accurate pollen interaction networks based on floral visitors. Convolvulaceae pollen in sediments have also been generally associated with canga vegetation and dry environment conditions along the Quaternary, ignoring the possible relationship with humid and forest environments as currently observed.

The pollen morphology of the Convolvulaceae has been analyzed by several researchers as an important taxonomic tool [19,20,21,22,23,24,25]. Nevertheless, palynological studies in South America are scarce [26,27]. Convolvulaceae is considered to be eurypalynous [28], with a classification based on a single character, which has caused uncertainty in its taxonomic classification [29]. Therefore, this study aims to present the detailed pollen morphology of Convolvulaceae species from the canga vegetation of Serra dos Carajás, based on light and scanning electron microscopy, to evaluate the potential of distinguishing their lower taxonomic levels and habitat types, which will improve the future studies on insect-plant interaction networks as well as paleoenvironmental analyses.

## 2. Materials and Methods

### 2.1. Study Area

**Figure 1:**
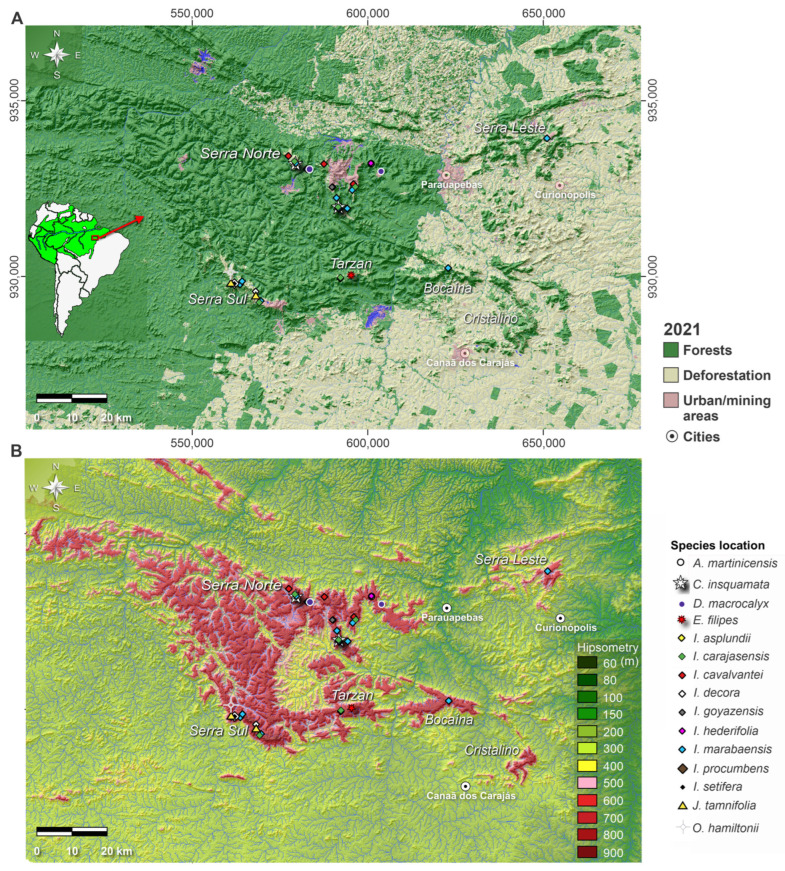
(**A**) Study site in the context of South America (red arrow) and Amazon basin. Source of metadata: Land Cover and Land Use layer of 2021 from MapBiomas Amazonia Project [37]; (**B**) Digital Elevation Model (SRTM, 30 m resolution) of the study site with the location of the Convolvulaceae species described in this work. The list of each species with geographic coordinates can be observed in Table S1.

Serra dos Carajás, located in the southeastern Amazonia, comprises the largest mineral province in Brazil and one of the largest in the world. In addition, this region is also home to a huge mosaic of conservation units protected by Brazilian legislation, which has protected the Amazon rainforest from conversion to pasture over the last fifty years (Figure 1A).

There are two vegetation types in Serra dos Carajás, humid evergreen tropical forests (HETF), which occur on the slopes of the plateau, interrupted by canga vegetation on the plateau at 600–800 m altitude (Figure 1B), which colonizes the lateritic crusts under edaphic conditions [15,30]. Several geoenvironments are described for canga areas, such as rupestrian and swampy fields, flat grasslands associated with sinkholes, active lakes, dry forests over degraded cangas, and open forests over aluminous-rich lateritic covers [31,32,33,34].

The cangas are areas with high species richness and unique floristic composition, including several endemic species that make the Carajás region an important area for the conservation of Amazonian flora [12].

The regional climate is tropical monsoon (Am; [35]). The average annual temperature is 26 °C. The rainfall regime is characterized by well-defined rainy (November to May) and dry (June to October) seasons with total annual rainfall of approximately 1700 and 240 mm, respectively [36].

### 2.2. Samples Collection and Slide Preparation for Morphological Descriptions

Occurrence of Convolvulaceae species at the study site is shown in Figure 1 and Table S1, and the examined specimens are stored in the herbaria of the Museu Paraense Emílio Goeldi (MPEG) and Parque Zoobotânico de Carajás (HCJS). Flower buds (mature) were extracted from the exsicatae collections and treated using standard pollen preparation methods including flower buds fixation in acetic acid, and acetolysis [38]. All slides were deposited in the Palinoteca of the Instituto Tecnológico Vale (PALIITV). For light microscopy (LM), the pollen was mounted in glycerol jelly, examined, measured, and photographed using a Zeiss AXIO Imager M2 microscope with a Pan-APOCHROMAT 20×, 40× and 100× objective. For scanning electron microspcopy (SEM), pollen grains were dehydrated with acetone, mounted on SEM stubs, coated with gold and imaged with a Zeiss Sigma VP microscope at 2000×, 6000×, 12,000× and 20,000× magnification. The following morphological parameters were measured: grain diameter (GDL and GDW), excluding echinae in *Ipomoea*; for the pores, the largest and smallest diameters (Pores_length and Pores_width) and distance between them (C_pores) were calculated. For the echinae, the base (Width_base_echinae) and height (Height_echinae), as well as the distance between them (DE) were measured. Measurement of exine stratification, composed of nexine (Nexine_thickness) and sexine (Sexine), was obtained at the interechinae region. The estimation of the number of echinae (Equation (1)) and pores per pollen grain (X_echinae and X_porus; Equation (2)) was based on [39]. These variables were examined in 20 grains per sample [40].

number of echinae = π·(equatorial diameter/interechinae distance)(1)

pores per pollen grain = π·(equatorial diameter/interpores distance)(2)

For the description of pollen morphology, the terminology used followed [41]. In the morphometric description, the data were organized in the following sequence: minimum value (standard deviation) and maximum value. For echinae, the classification was adapted from [42]: conical; bulbous with apiculate apex (type 1); bulbous with rounded apex (type 2); and bulbous with bulbous apex (type 3).

### 2.3. Statistical Analysis

Principal component analysis (PCA) was performed using 17 variables to assess whether the morphometric pollen characteristics allow the separation or clustering of species. The variables used for the analysis were as follows: largest grain diameter (GDL); smallest grain diameter (GDW); GDL/GDW ratio; largest porus diameter (Pores_length); smallest pores diameter (Pores_width); Pores_length/Pores_width ratio (Pl.Pw); C_pores/GDL ratio (C.GDL); C_pores/GDW ratio (C.GDW); distance between pores (C_pores); echinae base (Width_base_echinae); echinae height (Height_echinae); distance between echinae (DE); number of pores (X_pores); number of echinae (X_echinae); sexine (Sexine); nexine (Nexine_thickness) and exine.

The first three principal components with eigenvalues greater than 1 were considered. The results were presented in a biplot along the PC1 and PC2 axes. All analyses were performed using the statistical software R version 4.0.1 [43], and graphs were generated using ‘factoextra’ [44] and ‘corrplot’ [45].

## 3. Results

### 3.1. Pollen Morphology

Convolvulaceae pollen in this study are widely variable, but most of the genera can be well organized based on an apertural system with grains ranging from 3-colpate (*Cuscuta*, *Distimake* and *Operculina*), undef-pantocolpate (*Aniseia*), 12-pantocolpate (*Evolvulus*), 5-(4) colporate (*Jacquemontia*), and ~93–217 pantoporate (*Ipomoea*). Differentiation of pollen at the species level can be achieved using other morphological parameters, such as grain size classes, shape, and exine ornamentation (Table 1). All pollen descriptions can be observed below. Morphometric data of pollen grains are presented in Table 2.

*Aniseia* Choisy

*Aniseia martinicensis* (Jacq.) Choisy 

Figure 2A–D

Pollen grains are monads, large, apolar, radially symmetrical, circular; pantocolpate; tectate, sexine is much thicker than nexine in mesocolpus and decreases in thickness as it approaches the apertural region, columellate (Table 2). Exine ornamentation is microreticulate, heterobrochate. On SEM, exine ornamentation is microreticulate-microverrucate, microverrucae irregular in size and distribution.

*Cuscuta* L.

*Cuscuta insquamata* Yunck.

Figure 2E–H

Pollen grains are monad, medium, isopolar, radially symmetrical, subtriangulate, subprolate (Polar/Equatorial; P/E = 1.30); 3-colpate; tectate, columellate. Exine ornamentation is microreticulate. On SEM, exine ornamentation is microreticulate and microechinate, with granules inside the colpi.

*Distimake* Raf.

*Distimake macrocalyx* (Ruiz & Pav.) A.R. Simões & Staples

Figure 2I–L

Pollen grains are monad, large, isopolar, radially symmetrical, subtriangulate, subprolate (P/E = 1.32); 3-colpate; tectate, columellate, sexine is twice the thickness of nexine in the mesocolpus (Table 2), and decreases in thickness near the apertural region. Exine ornamentation is microgranulate and microreticulate on SEM, homogenously distributed in mesocolpium and apocolpium. Granules vary in size and shape and colpi are also granulated.

*Evolvulus* L.

*Evolvulus filipes* Mart.

Figure 2M–P

Pollen grains are monad, medium, apolar, radially symmetrical, circular; pantocolpate with 12 apertures; tectate, columellate, exine ornamentation is microreticulate. On SEM, exine ornamentation is microechinate.

*Ipomoea* L.

**Figure 2:**
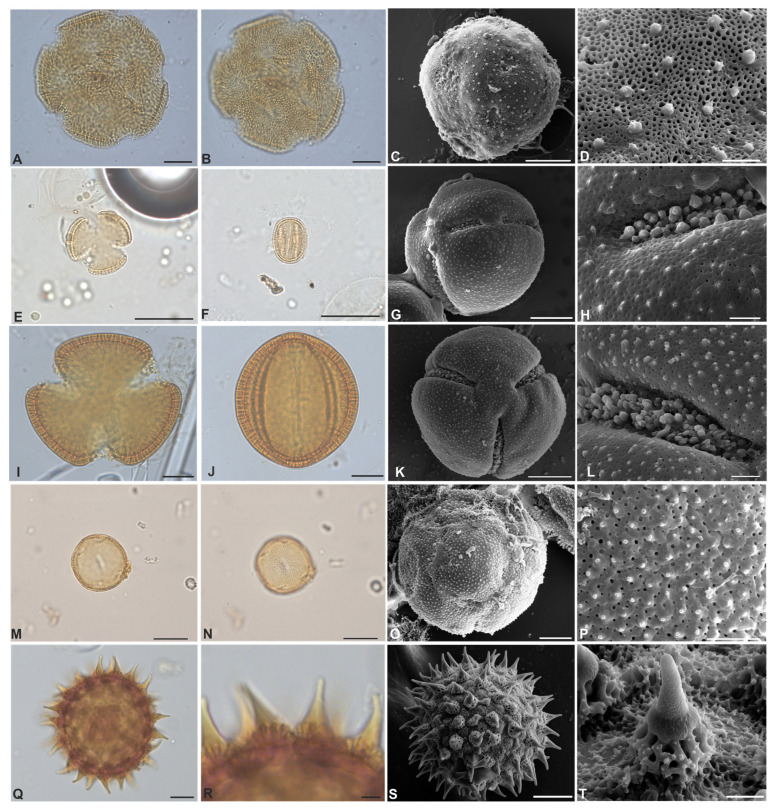
(**A**–**T**). Pollen grains of Convolvulaceae from Serra dos Carajás, Pará, Brazil. *Aniseia martinicensis* (**A**–**D**): (**A**)—optical section; (**B**)—ornamentation; (**C**)—surface (SEM); (**D**)—detail of the exine ornamentation; *Cuscuta insquamata* (**E**–**H**): (**E**)—optical section of the polar view; (**F**)—optical section of the equatorial view; (**G**)—surface (SEM); (**H**)—ornamentation detail of the exine and colpus; *Distimake macrocalyx* (**I**–**L**): (**I**)—optical section of polar view; (**J**)—optical section of equatorial view; (**K**)—surface on SEM; (**L**)—detail of the exine ornamentation and colpus; *Evolvulus filipes* (**M**–**P**): (**M**)—optical section; (**N**)—pantocolpate aperture; (**O**)—surface (SEM); (**P**,**Q**)—detail of the exine ornamentation; *Ipomoea asplundii* (**Q**–**T**): (**Q**)—optical section; (**R**)—conical echinae; (**S**)—surface SEM; (**T**)—echinus and detail of the columellae; (**A**–**C**,**E**–**G**,**I**–**K**,**M**–**O**,**Q**,**S**)—20-µm scale; (**R**)—5-µm scale; (**D**,**H**,**L**,**P**,**T**)—3-µm scale).

**Figure 3:**
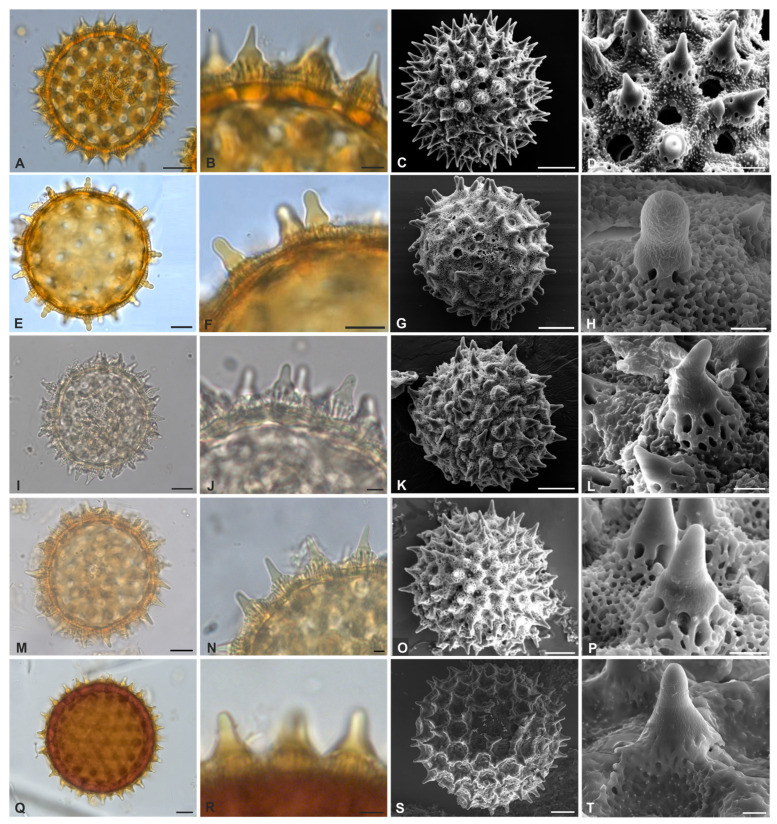
(**A**–**T**). Pollen grains of Convolvulaceae from Serra dos Carajás, Pará, Brazil. *Ipomoea carajasensis* (**A**–**D**) (**A**)—optical section; (**B**)—conical echinae; (**C**)—surface on SEM; (**D**)—echinae and apertures. *I. cavalcantei* (**E**–**H**): (**E**)—optical section; (**F**)—bulbous echinae type 3; (**G**)—Surface (SEM); (**H**)—detail of the echinae (SEM). *Ipomoea decora* (**I**–**L**) (**I**)—optical section; (**J**)—type 2 bulbous echinae; (**K**)—surface on SEM; (**L**)—echinae and detail of the columellae. *I. goyazensis* (**M**–**P**): (**M**)—optical section; (**N**)—type 1 bulbous echinae; (**O**)—surface on SEM; (**P**)—echinae and detail of columellae. *I. hederifolia* (**Q**–**T**): (**Q**)—optical section; (**R**)—type 2 bulbous echinae; (**S**)—surface on SEM; (**T**)—echinae and detail of the columellae. (**A**,**C**,**E**,**G**,**I**,**K**,**M**,**O**,**Q**,**S**)—20-µm scale; (**B**,**F**,**J**,**N**,**R**)—5-µm scale; (**D**,**H**,**L**,**P**,**T**)—3-µm scale).

**Figure 4:**
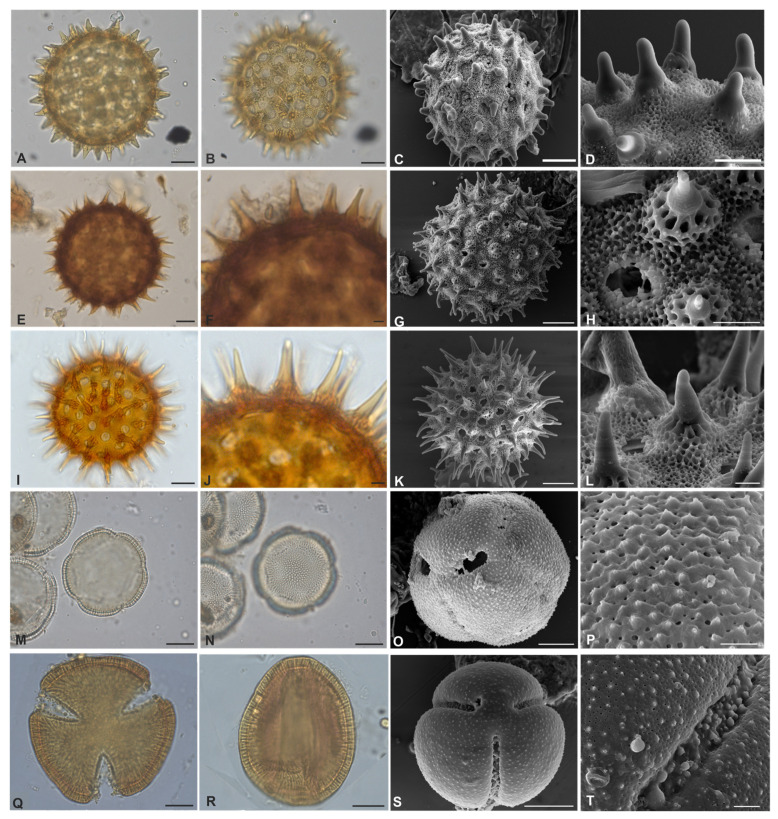
(**A**–**T**). Pollen grains of Convolvulaceae from Serra dos Carajás, Pará, Brazil. *I. marabaensis* (**A**–**D**): (**A**)—optical section; (**B**)—type 2 bulbous echinae; (**C**)—surface on SEM; (**D**)—echinae and detail of the columellae. *Ipomoea procumbens* (**E**–**H**) (**E**)—optical section; (**F**)—conical echinae; (**G**)—surface on SEM; (**H**)—echinae and detail of columellae and aperture. *Ipomoea setifera* (**I**–**L**): (**I**)—optical section; (**J**)—conical echinae; (**K**)—surface on SEM; (**L**)—echinae and detail of columellae. *Jacquemontia tamnifolia* (**M**–**P**): (**M**)—optical section; (**N**)—exine ornamentation on OM; (**O**)—surface on SEM; (**P**)—detail of the exine on SEM. *Operculina hamiltonii* (**Q**–**T**): (**Q**)-optical section of polar view; (**R**)—optical section of equatorial view; (**S**)—surface on SEM; (**T**)—detail of the exine ornamentation and colpus. (**A**,**B**,**C**,**E**,**G**,**I**,**K**,**M**,**N**,**O**,**Q**,**R**,**S**)—20-µm scale; (**F**,**J**)—5-µm scale; (**D**,**H**,**L**,**P**,**T**)—3-µm scale).

Figure 2Q–T, Figure 3A–T and Figure 4A–L

Pollen grains are monad, ranging from large to very large, with radial symmetry, apolar, circular, and pantoporate. The number of pores varies greatly among species ranging from ~93 to ~217, and they are circular to elliptical. Tectate to semitectate, columellate, and nexine is sometimes thinner than sexine, as in *Ipomoea goyazensis*. In all species, the macro-ornamentation is echinate, varying according to the echinae types (Table 1). The micro-ornamentation is microreticulate in the interechinae areas, with the presence of granules (Figure 2Q and Figure 3D,T). In *I. hederifolia*, echinae are evenly distributed around the pores, forming rosettes (Figure 3Q–T). Therefore, this genus may be considered stenopalynous.

*Jacquemontia* Choisy

*Jacquemontia tamnifolia* (L.) Griseb.

Figure 4M–P

Pollen grains are monad, medium to large, isopolar, radially symmetrical, circular; 5-(4) colporate; tectate, sexine is thicker than nexine in mesocolpus (Table 2), and decreases in thickness as it approaches the apertural region; columellate, exine ornamentation is microreticulate and microechinate; these elements are more easily visualized under SEM.

*Operculina* Silva Manso

*Operculina hamiltonii* (G.Don) D.F. Austin & Staples

Figure 4Q–T

Pollen grains monad, large, isopolar, radially symmetrical, subtriangulate, prolate spheroidal (P/E = 1.13); 3-colpate; tectate, sexine twice the thickness of nexine in the mesocolpus (Table 2), and decreases in thickness near the colpus; exine is microgranulate and microreticulate and easily visualized by SEM.

### 3.2. Statistical Analysis

**Figure 5:**
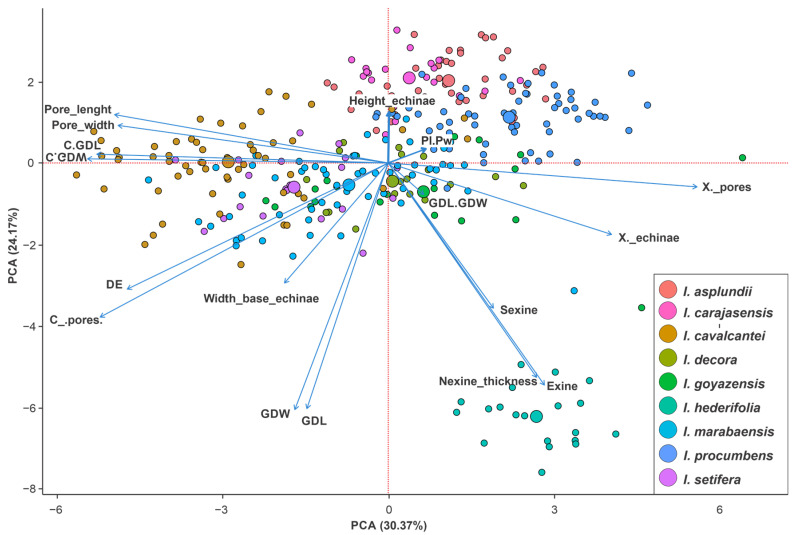
Principal component analysis scatterplot with the morphometric pollen variables of *Ipomoea*. The first two principal components were plotted, and each color represents a different species. Largest grain diameter (GDL); smallest grain diameter (GDW); GDL/GDW ratio; largest pores diameter (Pores_length); smallest pores diameter (Pores_width); Pores_length/Pores_width ratio (Pl.Pw); C_pores/GDL ratio (C.GDL); C_pores/GDW ratio (C.GDW); distance between pores (C_pores); echinae base (Width_base_echinae); echinae height (Height_echinae); distance between echinae (DE); number of pores (X_pores); number of echinae (X_echinae); sexine (Sexine); nexine (Nexine_thickness) and exine. The larger the dot, the greater the significance of the sample with the related parameter.

As observed on morphological analysis, the *Ipomoea* species are stenopalynous. In this case, an exploratory analysis of quantitative data based on the Principal Components Analysis (PCA) was used to analyze the pollen grains (Figure 5). This analysis was performed with 17 morphometric variables measured (Table 2). The first three axes of the analysis, with eigenvalues greater than 1, summarized 65% of the total data variance (Table S2).

The first major axis (PC1) was the most significant for species ordination (Figure 5), which explained 30.6% of the variation based mainly on the estimate of the number of pores (X._pores) and number of echinae (X._echinae), followed by the ratios of the distance between the pori and the largest grain size and smallest grain size (C.GDL and C.GDW), and the distance between pores (C. pores). PC2 was responsible for 24.17% of the data variability, mainly related to the grain size (GDL and GDW), exine thickness, and echinae height (Height_echinae). However, echinae height and width contributed negatively to the construction of the third axis (PC3), explaining 10.25% of the data variability.

In the first axis (Figure 5), *Ipomoea cavalcantei* has a larger distance between echinae (DE) values and a lower number of echinae and pores (X._echinae and X._pores). *I. procumbens* also has larger pores (Pl and Pw), distance between pores, and echinae base. The species *I. carajasensis*, *I. asplundii*, and *I. setifera* were grouped on the positive side of axes one and two, since they have higher echinae heights (Height_echinae), smaller exines and echinae bases (Width_echinae), and smaller pollen grains (GDL and GDW). In the second axis, *I. hederifolia* was grouped separately from the other species, with the main characteristic being the largest grains among species (GDL and GDW), with smaller pores (P and Pw) and higher exine, nexine, and sexine values. In addition*, I. hederifolia* had a higher number of echinae and pori calculated according to [38].

The pollen of *I. procumbens*, *I. setifera*, *I. asplundii*, *I. carajasensis*, *I. goyazensis*, *I. decora*, and *I. marabaensis* were clustered due to similar values of the Pl.Pw ratio and Height_echinae metrics (Figure 5).

## 4. Discussion

### 4.1. Palynotaxonomy

Among the species studied, *Cuscuta insquamata* and *Evolvulus felipes* have the smallest pollen grains, which may be related, but without generalizations, to the size of their flowers [50]. This morphological information can approximate the species and help in the taxonomic delimitation, because until the present study little was known about the *Cuscuta* [51]. *Evolvulus filipes* was described as pantocolpate with microechinate ornamentation on SEM, which corroborates with the descriptions of [24,55].

Considering the grouping of different pollen morphology characters, ref. [21] divided Convolvulaceae into four groups, as follows: Group 1, 5(-6)-colpate; Group 2, 3-colpate; Group 3, dodecacolpate; Group 4, pantoporate. According to this classification, *Cuscuta insquamata* belong to Group 2, which is characterized by medium-sized colpate pollen grains and the presence of granules inside the colpi. The *Ipomoea* species belongs to Group 4, due to the apertural type and its surface arrangement in the grains.

The nine *Ipomoea* species analyzed exhibit pollen grains with semitectate to tectate, pantoporate, and echinate exine, which corroborates the descriptions in several studies [20,21,24,26,42,47,48,49,52,53,54,56].

The echinae of *Ipomoea* are supported by thick columellae, which increase in height in the aperture-echinae direction, similar to the description by [24,52].

Echinae have been related as the main differentiating character of the *Ipomoea* species, defined according the base of the echinae, which ranges from straight to bulbar, and apex shape [47]. Therefore, they can be grouped in this study as follows: (a) conical (*I. asplundii*, *I. carajasensis*, *I. procumbens*, and *I. setifera*); (b) bulbous type 1, with bulbar base and apiculate apex (*I. goyazensis*); (c) bulbous type 2, with bulbar base and rounded apex (*I. decora*, *I. hederifolia* and *I. marabaensis*); d) bulbous type 3, with bulbous apex (*I. cavalcantei*). The sexine was thinner than the nexine in *I. hederifolia*, *I. marabaensis*, *I. procumbens*, and *I. setifera*. In addition, it presented the largest pollen grains with the largest diameter, ranging from 131–144 μm, classified as very large, corroborating with [50]. However, *I. hederifolia* does not fit with the data presented here, as they described the echinae as conical and located on edges, with bulbous echinae type 2 [53].

Intraspecific variations are observed, hindering the standardization of the morphological description, and are better detected in some species, such as *I. cavalcantei* and *I. marabaensis*. Likewise, it is difficult to establish the number of pores due to the density of the echinae and thick exine. The large number of apertures is possibly associated with derived taxa, and with greater reproductive efficiency due to increased opportunities for pollen tube germination [56].

Few diagnostic characters are known for *Jacquemontia*, resulting in identifications that are, in many cases, inaccurate. This is reflected in several botanical collections where the genus is erroneously identified as *Evolvulus* L. or *Convolvulus* L. [57,58]. However, the number of apertures and their distribution in the pollen grains helped in the differentiation of *Jacquemontia tamnifolia* and *Evolvulus filipes*.

New taxonomic combinations were presented by [59], with *Distimake* Raf. (=*Merremia* Dennst. ex Endl.). Due to this nomenclatural change based mainly on the phylogeny of the group, many palynological works prior to publication treated the species occurring in the cangas of Carajás as *Merremia macrocalyx* (Ruiz & Pav.) O’Donell.

*Distimake macrocalyx* has been erroneously described with psilate ornamentation [46]. In the SEM analysis, granulate and microreticulate exines were observed [60], which were classified as granules by [42]. *D. macrocalyx* has been described as having distally branched columellae [22]. The main differences between *D. macrocalyx* and *Operculina hamiltonii* are the size and shape, where *D. macrocalyx* has large and subprolate pollen grains, while *O. hamiltonii* has very large pollen grains with a spheroidal prolate shape. These findings corroborate with [61] and [42], but differ from those findings reported by [62], specifically related to *D. macrocalyx* (prolate grains). According to [26], 4-colpate pollen grains are found in *D. macrocalyx*, a characteristic that was not observed in the studied grains.

This study indicated that the qualitative characteristics of the echinae type, and in some cases, grain size and aperture arrangement, are important characteristics to describe the Convolvulaceae genera, thus establishing the classification of pollen types for the analyzed species. In addition, quantitative data (morphometry) confirm that the attributes used are suitable to classify the pollen types. Statistical methods such as PCA have been frequently used to evaluate the systematic utility of pollen data [63,64,65,66,67,68,69,70]. For example, *I. goyazensis* and *I. decora* presented conflicts in their taxonomic delimitations [71]. Indeed, we observed based on the PCA that the two species were grouped together (Figure 5), which may serve as evidence of their similarity.

The studied specimens of the genus *Ipomoea* were collected in different areas, which suggests that the geographical boundaries were not sufficient to result in a significant difference in pollen morphology, with the exception of *I. hederifolia*, which was grouped separately from the others. According to [72], closely related species generally produce similar pollen grains.

Some specimens, represented by same-color points on the PCA plot (Figure 5), are dispersed in the cluster, differing in the number of apertures in their pollen grains, such as *I. carajasensis.* This trend is often due to hybridization processes, and is linked to the level of ploidy in individuals [73,74,75].

### 4.2. Relevance for Interaction Networks and Paleoenvironmental Studies

The Convolvulaceae occurrence in Carajás cangas were based on a huge botanical survey and re-analysis of exsiccate by family specialists conducted by the Flora de Carajás Project [10,11]. Therefore, the family may be found in a wide variety of canga geoenvironments with multiple flower visitors [10,33].

Their flowers generally attract a wide range of visitors that include bees (melittophily) as the predominant group [76]. Indeed, from the studied species, only *Cuscuta insquamata*, *Ipomoea cavalcantei*, and *Ipomoea hederifolia* are ornithophilous [77]. This is a very interesting field observation for *C. insquamata*, since *Cuscuta* species usually have a large and varied court of visiting insects, including flies, moths, beetles, and predators such as spiders and larger insects [78]. *Ipomoea* flowers are tubular and should restrict entry into the floral tube only to visitors with adequate anatomy, thus characterizing a relationship between the size of the floral tube and the size of the visitor resulting in contact with reproductive structures, as a way to guarantee pollination [79]. Among the floral visitors of *Ipomoea* species, *Melitoma* bees are usually associated with their flowers [79,80,81,82]. *Trigona* bees are legitimate frequent visitors on *I. cavalcantei* and *I. marabaensis*. However, the predominant pollinators of *I. cavalcantei* are hummingbirds, according to [83]. The exine ornamentation in relation to pollinators has been described in reference [84]. Several plant species with microreticulated pollen grains are pollinated by bees [85,86,87]. This may be the case for *Aniseia martinicensis*, *C. insquamata*, *Evolulus filipes*, *Jacquemontia tamnifolia*, *Distimake macrocalyx*, and *Operculina hamiltonii*. The echinate surface is also a typical feature for pollen transfer by animals [84]. Indeed, echinae in *Ipomoea* pollen seem to allow pollen fixation to the hair of bees, optimizing the transport process [60]. Therefore, the pollen morphology is one of the factors responsible for the strengthening of the plant-pollinator relationship [88]. Convolvulaceae pollen in sediments were only identified at the genus level (i.e., *Ipomoea* and *Evolulus*), and were generally associated with canga vegetation and dry environment conditions along the Quaternary, ignoring the possible relationship with humid and forest environments, as currently observed [17,18,88]. In fact, this generalization is no longer acceptable. *Aniseia martinicensis*, *C. insquamata*, *Distimake macrocalyx*, *Evolvulus filipes*, *I. asplundii*, *I. carajasensis*, *I. cavalcantei*, *I. decora*, *I. goyazensis*, *I. hederifolia*, *I. marabaensis*, *I. procumbens*, *I. setifera*, *Jacquemontia tamnifolia* and *Operculina hamiltonii* are not exclusively found on rupestrian fields (Fe-Al lateritic crusts), but are also very common on swampy fields and borders of humid evergreen tropical forests (HETF), and dry and open forests, which suggests more humid habitat conditions and some protection from direct sunlight. In addition, the low classification level of *Ipomoea* and even *Jacquemontia* hinders the possible association with anthropic interventions in the natural environment. Indeed, *I. hederifolia*, *I. setifera*, and *J. tamnifolia* are frequently observed in altered areas as ruderal species [10].

## 5. Conclusions

These results reinforce the importance of studying pollen morphology to identify and distinguish genera and species of the family Convolvulaceae. Based on the data obtained, *Aniseia martinicensis* has large pantocolpate pollen with microreticulate/microverrucate ornamentation. A similar apertural type was found in *Evolvulus filipes*, but with medium-sized grains with microreticulate/microechinate ornamentation. *Cuscuta insquamata*, *Distimake macrocalyx*, and *Operculina hamiltonii* have 3-colpate pollen grains, but *C. insquamata* has medium-sized grains with microreticulate and microechinate ornamentation, while *D. macrocalyx* and *O. hamiltonii* have subprolate and prolate spheroidal grains, respectively. *Ipomoea* are pantoporate with echinate ornamentation. Indeed, echinae are the main differentiating character of the *Ipomoea* species, defined according to their basal morphology. However, apertural type and grain size must be used in conjunction to better classify each species. *I. asplundii*, *I. carajasensis*, *I. procumbens*, and *I. setifera* have conical echinae with ~163, 183, and 144 pores, respectively. *I. goyazensis* present bulbous type 1 echinae. *I. decora*, *I. hederifolia*, and *I. marabaensis* have type 2 bulbous echinae with ~160, 217, and 182 pores. *I. cavalcantei* has bulbous type 3 echinae. It is concluded that palynotaxonomy is considered an important and effective tool in species identification for taxonomic studies. This study of the Convolvulaceae taxa contributes to the knowledge on the Brazilian and worldwide pollen flora, and may contribute to taxonomic circumscription, and thus improve the understanding of the phylogenetic relationships of Convolvulaceae. In general, the set of morphological characters was effective for separating Convolvulaceae genera and species occurring in Serra dos Carajás and can be a reliable tool for future studies on insect-plant interaction networks as well as paleoenvironmental analyses.

## Figures and Tables

**Table 1:** Pollen morphology of Convolvulaceae species. M = medium, L = large; VL = very large. The size and shape classes follow [20]. FKD: First Known Description.

Species	Size	Shape	Aperture		Exine Ornamentation	Spine Type	Reference
			Type	No.			
*Aniseia martinicensis* (Jacq.) Choisy	L	circular	colpus	-	microreticulate and microverrucate	-	[24]
*Cuscuta insquamata* Yunck.	M	subprolate	colpus	3	microreticulate and microechinate	-	FKD
*Distimake macrocalyx* (Ruiz & Pav.) A.R.Simões & Staples	L	subprolate	colpus	3	microreticulate and microgranulate	-	[22,24,26,42,46,47,48,49]
*Evolvulus filipes* Mart.	M	circular	colpus	12	microreticulate and microechinate	-	FKD
*Ipomoea asplundii* O’Donell	L	circular	porus	~163	echinate and microreticulate with granules	Conical	FKD
*I. carajasensis* D.F. Austin	L	circular	porus	~183	echinate and microreticulate with granules	conical	[46]
*I. cavalcantei* DF Austin	L	circular	porus	~93	echinate and microreticulate with granules	bulbous type 3	[46]
*I. decora* Meisn.	L	circular	porus	~160	echinate and microreticulate with granules	bulbous type 2	FKD
*I. goyazensis* Gardner	VL	circular	porus	~199	echinate and microreticulate with granules	bulbous type 1	FKD
*I. hederifolia* L.	VL	circular	porus	~217	echinate and microreticulate with granules	bulbous type 2	[21,42,50,51,52,53,54]
*I. marabaensis* D.F. Austin & Secco	L/VL	circular	porus	~182	echinate and microreticulate with granules	bulbous type 2	[46]
*I. procumbens* Mart. ex Choisy	L/VL	circular	porus	~144	echinate and microreticulate with granules	conical	[42]
*I. setifera* Poir.	L/VL	circular	porus	~163	echinate and microreticulate with granules	conical	[24]
*Jacquemontia tamnifolia* (L.) Griseb.	F/M	circular	colpus/porus	5(4)	microreticulate and microechinate	-	[24,25,46]
*Operculina hamiltonii* (G.Don) D.F. Austin & Staple	L	prolate spheroidal	colpus	3	microreticulate and microgranulate	-	FKD

**Table 2:** Morphometry (µm) of pollen grains of Convolvulaceae species. PD = polar diameter (µm); ED = equatorial diameter (µm); LD = largest diameter of apolar grains; SD = smallest diameter of apolar grains; No = number, Di = interechinae distance (µm). * Presented as follows: minimum (standard deviation) maximum.

Species	PD/LD *	ED/SD *	Sexine *	Nexine *	Exine *	Spine			
						Base	Height	No.	Di
*Aniseia martinicensis*	82.7 (5.8) 108.2	76.5 (6.4) 99.9	4.3 (0.6) 6.4	1.2 (0.3) 2.4	6.0 (0.7) 11.6	-	-	-	-
*Cuscuta insquamata*	25.9 (6.4) 48.4	18.3 (4.3) 39.6	0.4 (0.1) 1.0	0.5 (0.1) 1.0	1.0 (0.5) 3.2	-	-	-	-
*Distimake macrocalyx*	69.0 (4.4). 86.6	83 (7.8) 72.2	2.9 (0.4) 4.7	0.8 (0.2) 1.8	3.9 (0.5) 6.0	-	-	-	-
*Evolvulus filipes*	28.2 (2.6) 36.0	25.7 (2.6) 33.9	0.6 (0.1) 1.0	0.8 (0.1) 1.1	1.4 (0.2) 1.9	-	-	-	-
*Ipomoea asplundii*	81.2 (5.5) 101.8	79.2 (5.0) 97.9	1.6 (0.4) 3.8	2.2 (0.4) 3.9	4.5 (0.6) 7.4	6.2	11.7	~185	13.0
*I. carajasensis*	83.6 (5.3) 111	82.2 (11.2) 101.2	2.8 (0.6) 6.1	2.8 (0.4) 4.8	6.4 (0.9) 9.8	5.1	8.7	~184	12.2
*I. cavalcantei*	92.7 (6.8) 125	85.2 (6.9) 125	2.9 (0.2) 3.5	2.3 (0.3) 3.2	5.4 (0.5) 6.8	6	8.9	~126	17.2
*I. decora*	98.6 (5.0) 116.3	87.4 (5.6) 112.2	2.7 (0.7) 5.6	2.6 (0.5) 4.7	6.4 (1.1) 10.0	6.2	8.7	~190	14.2
*I. goyazensis*	93.6 (12.2) 155.7	91.7 (8.5) 120.9	2.8 (0.6) 4.7	2.3 (0.5) 4.3	5.6 (0.9) 8.9	6.1	10.7	~197	14.9
*I. hederifolia*	131.2 (3.5) 144	126.7 (3.4) 140.7	4.0 (1.3) 9.2	4.6 (1.4) 10.9	11.8 (1.2) 16.6	6.7	9.0	~200	16.6
*I. marabaensis*	98.4 (8.8) 128	96.5 (6.5) 122	2.4 (0.5) 4.2	2.9 (0.3) 4.1	5.8 (0.6) 7.9	6.3	9.9	~185	15.2
*I. procumbens*	109.7 (4.2) 122.9	107.3 (2.9) 119.6	1.2 (0.8) 4.8	2.2 (0.6) 4.5	4.5 (1.2) 9.2	6.9	15.7	~175	15.8
*I. setifera*	99.1 (2.2) 100.5	87.5 (2.6) 99.1	1.4 (0.6) 3.5	2.5 (0.5)4.2	3.9 (0.8) 7.5	5.1	15.2	~147	13.9
*Jacquemontia tamnifolia*	42.9 (7.9) 69.1	41.6 (8.0) 65.7	1.8 (0.3) 3.3	0.6 (0.3) 1.6	2.5 (0.5) 4.8	-	-	-	-
*Operculina hamiltonii*	84.8 (9.7) 122	66.7 (9.8) 98.3	2.9 (0.7) 5.3	0.1 (0.4) 2.6	3.9 (0.7) 6.9	-	-	-	-

## Data Availability

All data are included in this manuscript.

## Supplementary Materials

The following supporting information can be downloaded at: https://www.mdpi.com/article/10.3390/plants12122256/s1: Table S1. Convolvulaceeae species described in this study; Table S2. Loadings of Ipomoea pollen morphological characters for three principal components. The first three PCs with eigenvalues greater than one are represented here. Each percentage in parentheses indicates the amount of variation explained by each PC. Largest grain diameter (GDL); smallest grain diameter (GDW); GDL/GDW ratio; largest pores diameter (Pores_length); smallest pores diameter (Pores_width); Pores_length/Pores_width ratio (Pl.Pw); C_pores/GDL ratio (C.GDL); C_pores/GDW ratio (C.GDW); distance between pores (C_pores); echinae base (Width_base_echinae); echinae height (Height_echinae); distance between echinae (DE); number of pores (X_pores); number of echinae (X_echinae); sexine (Sexine); nexine (Nexine_thickness) and exine.
