# Supplementary material for: Pollen Morphology of Convolvulaceae from Southeastern Amazonian Cangas and Its Relevance for Interaction Networks and Paleoenvironmental Studies
Source: Plants (Basel). 2023 Jun 9;12(12):2256. doi: 10.3390/plants12122256 (PMC10304759; doi:10.3390/plants12122256)
Supplement: Supplementary file 1 [file plants-12-02256-s001.zip › Table S1.pdf]

Table S1. Convolvulaceeae species described in this study.

Genera	Species	Ocorrence	Geographic coordinate	Herbarium Nº	Colector
Aniseia	*Aniseia cernua* Moric.	N5	6°02'30" S, 50°05'16" W	MG222501	Meirelles,J.968
Cuscuta	*Cuscuta insquamata* Yunck	N1	6º02'35" S, 50º16'57" W	MG 215432	Viana, P.L. 5594
		N7	6º09'16" S, 50º10'18" W	MG 215060	Mota, N.F.O. 3434
Evolvulus	*Evolvulus filipes* Mart	Serra do Tarzan	6°19'44" S, 50°08'20" W	MG 214460	Mota, N.F.O. 3003
Ipomoea	*Ipomoea asplundii* O'Donell	N7	6°09'29" S, 50°10'10" W	MG 226331	Vasconcelos, L.V. 1102
		N7	6°09'29" S, 50°10'14" W	MG 222398	Harley, R.M. 57379
	Ipomoea cavalcantei D.F.Austin	N1	6°01'20" S, 50°18'02" W	MG 223584	Falcão, B.F. 182
		N2	6º03'21" S, 50º15'13" W	MG 215469	Viana, P.L. 5631
		N3	6º02'34" S, 50º12'33" W	MG 214408	Mota, N.F.O. 2951
		N4	6°06'08" S, 50º11'13" W	MG 223179	Harley, R.M. 57491
		N5	6°05'40" S, 50°07'59" W	IAN 173120	L.V. Vasconcelos
	Ipomoea decora Meisn.	Estrada S11D A S11A	6º22'17" S, 50°23'04" W	MG 213191	Lobato, L.C.B. 4406
	Ipomoea goyazensis Gardner	N4	-	MG 227195	L.V. Vasconcelos

	Ipomoea hederifolia L.	N5	6º02'26" S, 50º05'18" W	HCJS 1224	Silva, D. F. & Tyski, 773
	Ipomoea marabaensis D.F.Austin & Secco	N1	6º02'27" S, 50º16'54" W	-	-
		N2	6°03'25" S, 50°15'04" W	-	-
		N5	6°06'35" S, 50°08'11" W	MG 223537	Falcão, B.F. 135
		N6	6°07'48" S, 50°10'36" W	MG 223639	Falcão, B.F. 237
		N7	6°09'25" S, 50°10'19" W	BHCB 162046	Arruda, A. J. 1366
		N8	6°09'26" S, 50°08'56" W	BHCB 162045	Arruda, A. J. 1365
		S11A	6°21'11" S, 50°25'30" W	BHCB 140215	Costa, F. M. 100
		S11B	6°20'42" S, 50°25'10" W	MG 223488	Falcão, B.F. 86
		S11D	6°23'44" S, 50°22'17" W	MG 213954	Carreira, L.M.M. 3337
		Serra da Bocaina	6°18'36" S, 49°53'20" W	MG 223758	Falcão, B.F. 356
		Serra Leste	5°58'32" S, 49°38'07" W	HCJS 5000	-
		Serra do Tarzan	6°20'15" S, 50°10'00" W	BHCB 130853	Giorni, V. T. 144
	Ipomoea maurandioides Meisn	N1	6°02'10" S, 50°17'06" W	MG 224469	Pastore, M. 343

		N5	6º06'05" S, 50º07'42" W	MG 214372	Mota, N.F.O. 2915
		N7	6º09'13" S, 50º10'21" W	BHCB 157742	Arruda, A. J. 858
		S11D	6°23'58" S, 50°22'31" W	MG 214004	Carreira, L.M.M. 3387
		S11A	6°21'09" S, 50°26'54" W	MG 214059	Carreira, L.M.M. 3442
		Serra do Tarzan	6°20'06" S, 50°09'58" W	MG 223299	Vasconcelos, L.V. 845
	Ipomoea procumbens Mart. ex Choisy	S11B	6°21'04" S, 50°26'22" W	MG 222344	Carreira, L.M.M. 3527
	Ipomoea setifera Poir.	Parque	6°03'39" S, 50°03'43" W	-	Rodrigues, T.
Jacquemontia	*Jacquemontia tamnifolia* (L.) Griseb.	S11C	6°23'06" S, 50°23'03" W	MG 223119	Harley, R.M. 57430
		S11D	6°21'09" S, 50°26'54" W	MG 214054	Carreira, L.M.M. 3437
Merremia	*Merremia macrocalyx* (Ruiz & Pav.) O'Donell	N2	6º03'23' S, 50º14'46" W	MG 215937	Lobato, L.C.B. 4447
		Parque	6º03'42" S, 50º03'44" W	-	Rodrigues, T.
Operculina	*Operculina hamiltonii* (G.Don) D.F.Austin & Staples	Estrada para Serra Sul	6º19'15” S, 50º26'58" W	HCJS 2045	Tyski, L. 198
