# Supplementary material for: Pollen Morphology of Convolvulaceae from Southeastern Amazonian Cangas and Its Relevance for Interaction Networks and Paleoenvironmental Studies
Source: Plants (Basel). 2023 Jun 9;12(12):2256. doi: 10.3390/plants12122256 (PMC10304759; doi:10.3390/plants12122256)
Supplement: Supplementary file 1 [file plants-12-02256-s001.zip › Table S2.pdf]

Table S2. Loadings of *Ipomoea* pollen morphological characters for three principal components. The first three PCs with eigenvalues greater than one are represented here. Each percentage in parentheses indicates the amount of variation explained by each PC. Largest grain diameter (GDL); smallest grain diameter (GDW); GDL/GDW ratio; largest pores diameter (Pores_length); smallest pores diameter (Pores_width); Pores_length/Pores_width ratio (Pl.Pw); C_pores/GDL ratio (C.GDL); C_pores/GDW ratio (C.GDW); distance between pores (C_pores); echinae base (Width_base_echinae); echinae height (Height_echinae); distance between echinae (DE); number of pores (X_pores); number of echinae (X_echinae); sexine (Sexine); nexine (Nexine_thickness) and exine.

**Characters**	**PC1 (30.6%)**	**PC2 (24.17%)**	**PC3 (10.25%)**
GDL	-0.0968558487	-0.4448954923	-0.2094374965
GDW	-0.1111947746	-0.4466275440	-0.1449467515
GDL.GDW	0.0422884533	-0.0570466784	-0.2967254499
Pores_length	-0.3257246993	0.0875787758	-0.2761904120
Pores_width	-0.3215687248	0.0680506747	-0.2752009761
Pl.Pw	0.0446138575	0.0255071817	0.0460258691
C_pores	-0.3429523932	-0.2791425832	0.0095841515
C.GDL	-0.3469113525	0.0158739705	0.2663134002
C.GDW	-0.3578827122	0.0074901452	0.2482642165
Width_base_echinae	-0.1234947500	-0.2173683353	-0.3158851823
Height_echinae	0.0009557029	0.0921775804	-0.4765036952
DE	-0.3106893994	-0.2284124864	0.0206751589
X_echinae	0.2658107702	-0.1293933233	-0.2076926665
X_pores	0.3675813652	-0.0432977352	-0.2368559242
Nexine_thickness	0.1765245315	-0.3882438747	0.0903857449
Sexine	0.1251662916	-0.2628771862	0.2887846217
EXINE	0.1858772727	-0.4022241713	0.2054888818
